# Sequence and phylogenetic analysis of the chloroplast genome for *Rosa xanthina*

**DOI:** 10.1080/23802359.2020.1792369

**Published:** 2020-07-20

**Authors:** Chengwen Gao, Chuanhong Wu, Qian Zhang, Mingxuan Wu, Ruirui Chen, Yalin Zhao, Aiguo Guo, Zhiqiang Li

**Affiliations:** The Affiliated Hospital of Qingdao University & The Biomedical Sciences Institute of Qingdao University (Qingdao Branch of SJTU Bio-X Institutes), Qingdao University, Qingdao, China

**Keywords:** *Rosa xanthina*, Manchu rose, Illumina sequencing, chloroplast genome

## Abstract

*Rosa xanthina* is a wild shrub rose that is native to open slopes and scrubby areas in central China. In this study, the chloroplast genome sequence of *R. xanthina* been assembled and characterized by Illumina sequencing. The complete chloroplast genome was 157,214 bp in length, containing a large single-copy region (LSC) of 86,302 bp and a small single-copy region (SSC) of 18,770 bp, which were separated by a pair of 26,071 bp inverted repeat regions (IRs), each. A total contained 137 genes, including 90 protein-coding, 8 rRNA, and 39 tRNA genes. Maximum-likelihood phylogenetic analysis of 15 representative plastomes within the order *Rosa* suggests that *R. xanthina* closely related to the *Rosa berberifolia*.

Rosaceae, containing about 3000 species in over 100 genera, is the third most economically significant plant family in temperate regions (Potter et al. 2007). The genus *Rosa* comprises about 200 species, *Rosa xanthina*, commonly called Manchu rose, is a wild shrub rose that is native to open slopes and scrubby areas in central China (Wang et al. 2012). The nutrients, such as amino acid, phospholipids, glucose oxidase DNA, and RNA, in *R. xanthina* are rich and the proportion is good (Zhi et al. 2002; Wang et al. 2016). *Rosa xanthina* has a potential value of application. In this study, we characterized the complete chloroplast genome sequence of *R. xanthina* as a resource for future genetic studies on related species.

Total DNA (Voucher specimen: N48.85°, E2.35°, INRA) was isolated using a modified CTAB method (Allen et al. 2006) and sequenced by the Illumina HiSeq 2500 (Illumina Inc., San Diego, CA) platform with pair-end (2 × 300 bp) library. generating approximately 16.1 GB of sequence data. Raw sequencing data were filtered using Trimmomatic 0.38 (Bolger et al. 2014), and the resulting clean data were used for assembly. De novo assembly using SPAdes 3.61 (Bankevich et al. 2012) with different K-mer parameters. De novo scaffolds with a positive relation to chloroplasts were ordered on to the reference chloroplast genome of *R. chinensis* (CM009590). Paired-end reads were remapped to consensus assembly with multiple iterations to fill gaps in final consensus sequence that was performed on Geneious Prime software version 2020.0.4 (Kearse et al. 2012). Chloroplast genome annotation using GeSeq to predict genes encoding proteins, transfer RNA (tRNA) and ribosomal RNA (rRNA), and adjusted manually as needed (Tillich et al. 2017). We manually examined the IR junctions of *R. xanthina*. The accurate new annotated complete chloroplast genome was submitted to GenBank with accession number MT547539.

The total plastome length of *R. xanthina* was 157,214 bp, with a large single-copy (LSC; 86,302 bp), small single-copy (SSC; 18,770 bp), and two inverted repeats (IRa and IRb; 26,071 bp, each). The overall GC content was 37.20% and the plastome contained 137 genes, including 90 protein-coding, 8 rRNA, and 39 tRNA genes. Among them, 39 genes are related to photosynthesis and 26 genes are involved in self-replication.

To further investigate its taxonomic status, a maximum-likelihood (ML) tree was constructed based on complete chloroplast genome sequences using MEGA 7.0 (Kumar et al. 2016) with 1000 bootstrap replicates. Here, the sequences of *Rosa* chloroplast genomes were aligned using MAFFT 7.221 (Katoh and Standley 2013). Chloroplast genome sequences of 15 species in *Rosa* (*R. banksiae*, *R. berberifolia*, *R. canina*, *R. chinensis*, *R. chinensis* var. *spontanea*, *R. hybrid cultivar*, *R. laevigata*, *R. laevigata* var. *leiocarpa*, *R. lucieae*, *R. multiflora*, *R. praelucens*, *R. rugosa*, *R. maximowicziana*, *R. roxburghii*, *R. xanthina* and *Geum rupestre* as the outgroup (Figure 1). The chloroplast genome of *R. xanthina* can contribute to our understanding of the phylogeny and evolution of this species.

**Figure 1.**
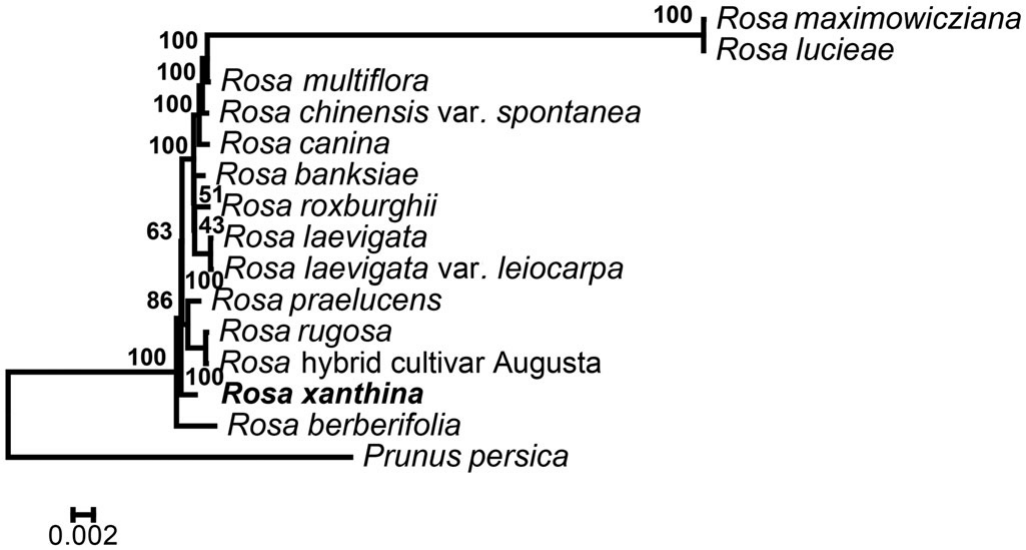
Phylogenetic relationships of 15 species based on chloroplast genome sequences. Taxon in bold formatting is the new chloroplast genome reported in this study. Bootstrap support is indicated for each branch. GenBank accession number: *R. banksiae* (NC_042194)*, R. berberifolia* (NC_045126), *R. canina* (NC_047295), *R. chinensis* (CM009590), *R. chinensis* var. *spontanea* (NC_038102), *R. hybrid cultivar* (NC_044126), *R. laevigata* (NC_046824), *R. laevigata* var. *leiocarpa* (NC_047418), *R. lucieae* (NC_040997), *R. multiflora* (NC_039989), *R. praelucens* (NC_037492), *R. rugosa* (NC_044094), *R. maximowicziana* (NC_040960), *R. roxburghii* (NC_032038), *R. xanthina* (MT547539), *Geum rupestre* (NC_037392).

## Data Availability

The data that support the findings of this study are openly available in GenBank of NCBI at https://www.ncbi.nlm.nih.gov, reference number MT547539.
